# Altered regulation of metabolic pathways in human lung cancer discerned by ^13^C stable isotope-resolved metabolomics (SIRM)

**DOI:** 10.1186/1476-4598-8-41

**Published:** 2009-06-26

**Authors:** Teresa WM Fan, Andrew N Lane, Richard M Higashi, Mohamed A Farag, Hong Gao, Michael Bousamra, Donald M Miller

**Affiliations:** 1 Department of Chemistry, University of Louisville, Louisville, Kentucky 40292, USA; 2 James Graham Brown Cancer Center, University of Louisville, Louisville, Kentucky 40292, USA; 3 Department of Surgery, University of Louisville, Louisville, Kentucky 40292, USA; 4 Center for Regulatory and Environmental Analytical Metabolomics (CREAM), University of Louisville, Louisville, Kentucky 40292, USA; 5 Pharmacognosy Department, Faculty of Pharmacy, Kasr El Aini St, P.B. 11562, Cairo University, Egypt

## Abstract

**Background:**

Metabolic perturbations arising from malignant transformation have not been systematically characterized in human lung cancers *in situ*. Stable isotope resolved metabolomic analysis (SIRM) enables functional analysis of gene dysregulations in lung cancer. To this purpose, metabolic changes were investigated by infusing uniformly labeled ^13^C-glucose into human lung cancer patients, followed by resection and processing of paired non-cancerous lung and non small cell carcinoma tissues. NMR and GC-MS were used for ^13^C-isotopomer-based metabolomic analysis of the extracts of tissues and blood plasma.

**Results:**

Many primary metabolites were consistently found at higher levels in lung cancer tissues than their surrounding non-cancerous tissues. ^13^C-enrichment in lactate, Ala, succinate, Glu, Asp, and citrate was also higher in the tumors, suggesting more active glycolysis and Krebs cycle in the tumor tissues. Particularly notable were the enhanced production of the Asp isotopomer with three ^13^C-labeled carbons and the buildup of ^13^C-2,3-Glu isotopomer in lung tumor tissues. This is consistent with the transformations of glucose into Asp or Glu via glycolysis, anaplerotic pyruvate carboxylation (PC), and the Krebs cycle. PC activation in tumor tissues was also shown by an increased level of pyruvate carboxylase mRNA and protein.

**Conclusion:**

PC activation – revealed here for the first time in human subjects – may be important for replenishing the Krebs cycle intermediates which can be diverted to lipid, protein, and nucleic acid biosynthesis to fulfill the high anabolic demands for growth in lung tumor tissues. We hypothesize that this is an important event in non-small cell lung cancer and possibly in other tumor development.

## Background

Uncontrolled growth is a universal trait of tumor cells, which requires profound changes in cellular metabolism to sustain the additional energy and biosynthetic precursor demands of proliferation. Accelerated aerobic glycolysis represents one such trait of malignant transformation, which was first described more than 80 years ago (i.e. the Warburg Effect) [1]. Activation of glycolysis in human lung cancers and cancer cells was inferred from an up-regulation of glycolysis-related enzymes such as hexokinase II (HK II), glyceraldehyde-3-phosphate dehydrogenase (GAPDH), and the bifunctional regulatory enzyme phosphofructokinase 2 (PFK-2) [2-7]. These enzyme expression changes associated with glucose oxidation are accompanied by an up-regulation of glucose transporters (e.g. GLUT 1) in non-small-cell lung cancers (NSCLC) [8]. Such up regulation associated with glucose metabolism is a hallmark of other human cancers as well.

Despite the dramatic glycolytic up regulation in many cancer cells, this process alone is insufficient to provide the necessary precursors for anabolic metabolism, so they must be supplied by additional metabolic processes. One key source of anabolic precursors is the Krebs cycle. Several of the Krebs cycle metabolites, such as citrate, oxaloacetate/aspartate, and α-ketoglutarate/glutamate are respective precursors for the biosynthesis of fatty acids, nucleic acids and proteins [9], all of which are required for growth. As some of these metabolites (e.g. oxaloacetate and α-ketoglutarate) are kept low in their cellular concentration, they will have to be replenished via anaplerosis to sustain both Krebs cycle and biosynthetic activities. This can be achieved by two anaplerotic pathways involving pyruvate carboxylation [10] and glutaminolysis [11]. The relative importance of these two pathways appears to be tissue specific [10,12,13].

To facilitate the search for key metabolic transformation processes in tumors, a systematic determination of tumor-specific metabolic alterations is crucially needed, particularly in terms of *in situ *human studies. Because the technology to perform these studies has been lacking, our current knowledge of lung cancer metabolism is limited and largely inferred from gene or protein expression changes, as described above [14]. Although gene/protein expression events provide useful clues to metabolic dysfunctions in lung cancer, they may not give a complete picture of metabolic changes that result in the malignant phenotype. It is clear that posttranslational modifications, protein inhibitors, allosteric regulation by effector metabolites, alternative gene functions, or compartmentalization also elicit important metabolic changes. Therefore, metabolic profiling (or metabolomic) investigations that complement transcriptomic and proteomic studies are essential to complete a systems biochemical understanding of malignant phenotypes.

The technological demand for metabolomic analysis is being met by recent advances in NMR spectroscopy and mass spectrometry (MS). Using these two complementary analytical platforms, it is now practical to simultaneously identify and quantify a large number of metabolites directly in crude extracts without the need for fractionation [15-20]. For example, metabolite identification in crude mixtures has been greatly accelerated by employing two-dimensional (2-D) NMR techniques such as 2-D ^1^H TOCSY (total correlation spectroscopy) and ^1^H-^13^C HSQC (heteronuclear single quantum coherence spectroscopy). TOCSY traces intramolecular interactions between protons through the covalent network, establishing it as a powerful tool for identifying molecular structures. HSQC complements TOCSY by detecting one-bond linkages between ^1^H and ^13^C within the molecular framework of metabolites. With the aid of an extensive NMR metabolite database, these two experiments together can unequivocally identify many metabolites in a complex mixture [20,21]. At this early stage of application to lung metabolism, a few metabolomic studies have demonstrated the utility of NMR and MS in providing global metabolite profiles in lung cells and tissues, bronchioalveolar lavage fluids (BALF), and urine from model animals engrafted with lung cancers [22-25]. However, none has been conducted on lung cancer tissues resected from patients.

In addition to providing metabolite profiles, NMR and MS are excellently suited for the analysis of labeling patterns of individual atoms (i.e. isotopomers) in a wide range of metabolites when conducting stable isotopic tracer studies. For example, 2-D ^1^H TOCSY and ^1^H-^13^C HSQC experiments profile ^13^C enrichment at specific atomic positions (i.e. ^13^C-positional isotopomers) while MS analyses enable quantification of ^13^C-mass isotopomers, regardless of the labeled position(s) [19]. Such ^13^C labeling profiles are crucial to simultaneously tracing multiple biochemical pathways without the challenging effort required in the past [13,26-30]. We have recently used uniformly labeled ^13^C glucose to trace the transformation of individual glucose carbons into a variety of metabolites via glycolysis, Krebs cycle, pentose phosphate pathway (PPP), glutathione (GSH) biosynthesis, and nucleotide biosynthesis in human lung adenocarcinoma A549 cells [22]. These metabolic transformation processes were corroborated by transcriptomic analysis [22,31].

Here we have extended the application of such ^13^C-tracer and isotopomer approaches to human lung cancer patients. The stable isotope tracer [U-^13^C]-glucose was administered intravenously into recruited patients prior to surgical resection of the primary tumor and surrounding non-cancerous tissues. Differences in metabolic pathways between paired non-cancerous lung and cancer tissues were profiled using NMR and MS. This approach enabled analysis of metabolic traits of cancer tissues without interferences from either intrinsic (e.g. genetic) or external environmental factors (e.g. diet) because patient's own non-cancerous tissue served as internal control. Compared to non-cancerous lung tissues, lung tumors demonstrated an enhanced capacity for glycolysis while displaying other distinct metabolic activities either unexpected or previously unknown. In particular, lung tumor tissues exhibited an altered but full Krebs cycle activity and enhanced pyruvate carboxylation, which could play a pivotal role in replenishing anabolic precursors required by tumor growth. This anabolic trait could also be fundamental to other human cancers.

## Results

### Time course analysis of ^13^C-Metabolites in plasma

**Figure 1:**
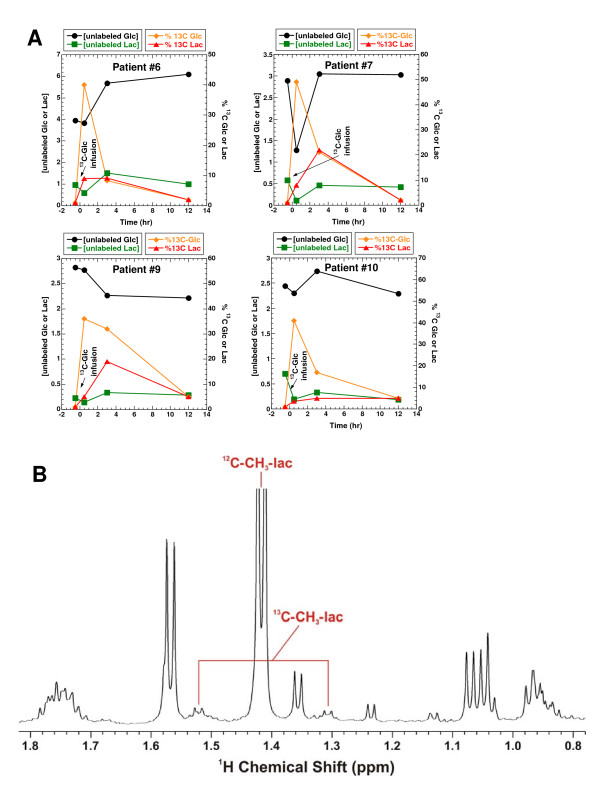
**Plasma samples were collected 0, 3 and 12 hr after [U-^13^C-Glc] infusion, as described in the Experimental Section**. Unlabeled glucose and lactate concentrations were quantified by 1-D ^1^H NMR using DSS as the calibration standard. Their % ^13^C enrichment was determined from the respective ^13^C satellite peaks in 1-D ^1^H NMR spectra. Panel A illustrates the time course changes in glucose and lactate concentrations as well as their % ^13^C enrichment. Panel B shows the ^13^C satellite pattern of the 3-methyl group of lactate (^13^C-CH_3_-lac) in the 1-D ^1^H NMR spectrum of patient #6 after 3 hr of [U-^13^C-Glc] infusion. The chemical shifts of lactate and Ala reflected the acidic pH of the TCA extract.

**Figure 2:**
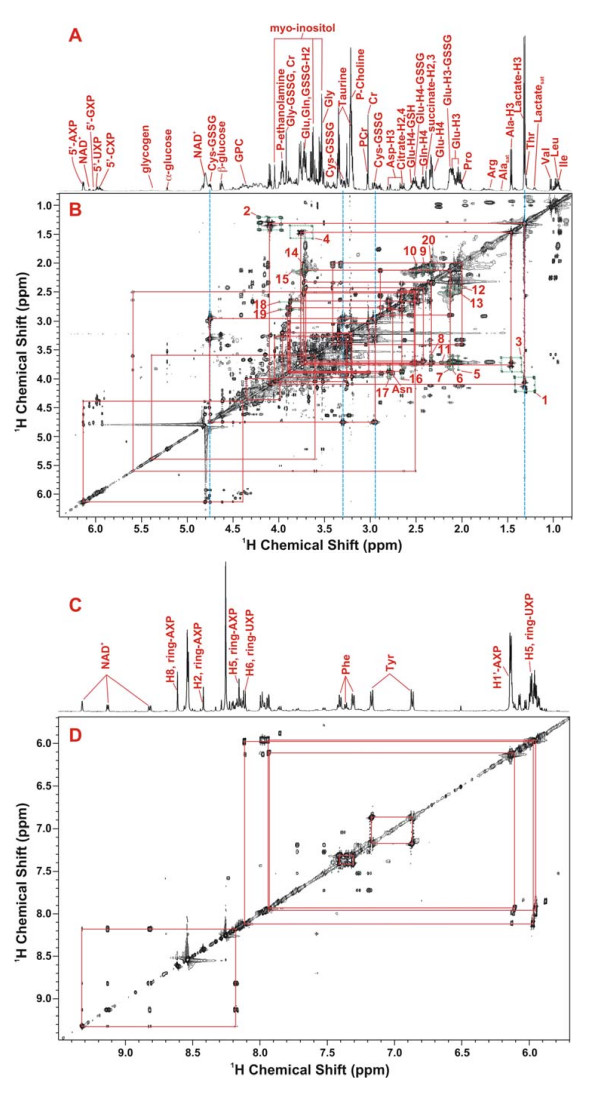
**2-D ^1^H TOCSY identification of polar metabolites in the lung tumor tissue of patient #6**. The 2-D TOCSY contour map is displayed along with the corresponding 1-D high-resolution spectrum. Panels A, B and C, D show the 0.8–6.4 and 5.7–9.5 ppm regions of the spectra, respectively. The assignment of cystine residue of oxidized glutathione (GSSG) was illustrated, which was based on the ^1^H covalent connectivity (traced by solid rectangles) and chemical shifts (traced by dashed blue lines). Lactate was discerned similarly and based on the peak splitting pattern (doublet for 3-methyl @ 1.32 ppm and quartet for 2-methine protons @ 4.11 ppm). In addition, the ^13^C satellite cross-peaks of 3-methyl and 2-methine protons of lactate (patterns 1 and 2) and Ala (patterns 3 and 4) were evident (traced by dashed green rectangles), and the peak pattern indicates that lactate and Ala were uniformly ^13^C labeled [19]. The ^13^C satellite cross-peak patterns for the protons of Glu (5, 6, 12, 20), Gln (9), glutamyl residue of oxidized glutathione (GSSG) (7, 10, 13) and Asp (16–19) were present and noted by vertical and horizontal dashed green lines. The ^13^C satellite cross-peaks 14 and 15 were contributed by a mixture of ^13^C-2-Glu, ^13^C-2-Gln, and ^13^C-2-Glu of reduced glutathione (GSH).

In order to determine the optimal time to sample tissue after infusing [U-^13^C]-Glc into lung cancer patients, we used ^1^H NMR to analyze the profiles of ^13^C-labeled products in plasma samples at several time points. Other than the administered tracer itself ([U-^13^C]-Glc), the major ^13^C-labeled metabolite in plasma was lactate. The time course changes in glucose and lactate concentrations and their % ^13^C enrichment are shown in Figure 1A. Plasma glucose was maximally enriched (up to 49%) in ^13^C immediately (0.5 hr) following the tracer infusion. Three hours later, a significant fraction (8.3–32%) of the plasma glucose remained ^13^C-labeled but by 12 hrs, the % ^13^C enrichment dropped to 2–5%, as illustrated for four patients. The % ^13^C-labeled lactate showed a similar time course as the % ^13^C-labeled glucose, except that the % enrichment reached a maximum (5–22%) after 3 hrs of infusion (Figure 1A). These data show that once taken up by tissues, ^13^C-glucose was metabolized quickly to ^13^C-lactate and secreted back into the blood. In addition, a large fraction of the plasma ^13^C-lactate was uniformly labeled in ^13^C, as evidenced by the fine splitting structure of the ^13^C satellites of the 3-methyl protons of lactate (Figures 1B and 2) [19,20] and by GC-MS analysis (Table 1). GC-MS analysis also revealed a significant presence of mass isotopomers of lactate with one or two ^13^C labels for patients #8–10, which is consistent with an active Cori cycle [9]. Based on this time-course analysis of ^13^C-isotopomers of metabolites in human plasma samples, we chose a duration of 3–4 h between ^13^C-glucose infusion and surgical resection for patients #6–10 in order to optimize ^13^C incorporation from [U-^13^C]-Glc into various metabolites.

**Table 1:** Percentage distribution of ^13^C-mass isotopomers of lactate in the plasma of human lung cancer patients infused with [U-^13^C]-glucose for 3 h^A^

	lactate+1^B^	lactate+2^B^	lactate+3^B^
#6	0.80%	0.36%	1.89%
#7	0.00%	1.83%	4.84%
#8	2.94%	0.16%	3.31%
#9	0.00%	4.11%	3.47%
#10	2.01%	1.00%	6.37%

^A ^Values in excess of natural abundance as determined from GC-MS analysis

^B ^Lactate+1, 2, 3 respectively refer to lactate with one, two, and three carbons labeled in ^13^C.

Of the twelve subjects undergoing ^13^C glucose infusion, the median age was 63 (range 52–76), 67% were male, 67% had squamous cell carcinoma, and 33% adeno- or adenosquamous carcinoma, all of grade II or III. All subjects had a history of heavy smoking.

### Metabolite &^13^C-Isotopomer profiling of lung tissue extracts by NMR

**Figure 3:**
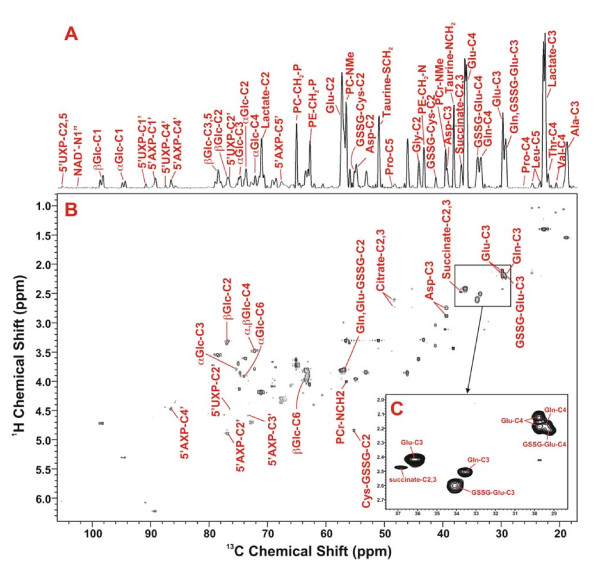
**^1^H-^13^C 2-D HSQC identification of ^13^C-metabolites in the TCA extracts of lung tumor tissues of patient #6**. Metabolites were identified based on ^1^H-^13^C covalent linkages observed in the 2-D contour map (panel B) and from the TOCSY spectrum such as in Fig. 2B. Panel A is the 1-D projection spectrum of the 2-D data along the ^13^C dimension, which allows a better comparison of the peak intensity of different metabolites. Panel C displays the expanded spectral region of C3 and C4 resonances of Glu, Gln, and GSSG-Glu to illustrate the resolution of these resonances in the 2-D HSQC contour map.

An example of the assignment of metabolites and their ^13^C-isotopomers in the TCA extracts by 2-D ^1^H TOCSY and high-resolution 1-D NMR spectra is illustrated in Figure 2. Assignments were made by matching the chemical shifts, spin-spin coupling patterns, and available covalent connectivities (traced by solid red rectangles in the TOCSY contour maps) of individual resonances against those of the standard compounds [20,21]. Metabolites that were commonly observed in all lung tissue extracts include isoleucine (Ile), leucine (Leu), valine (Val), lactate, alanine (Ala), arginine (Arg), proline (Pro), glutamate (Glu), oxidized glutathione (GSSG), glutamine (Gln), succinate, citrate, aspartate (Asp), creatine (Cr), phosphocholine (P-choline), taurine, glycine (Gly), phenylalanine (Phe), tyrosine (Tyr), myo-inositol, α- and β-glucose, NAD^+^, cytosine nucleotides (CXP), uracil nucleotides (UXP), guanine nucleotides (GXP), and adenine nucleotides (AXP). The ^1^H TOCSY assignment of metabolites was complemented by the 2-D ^1^H-^13^C HSQC analysis of the same extract, as shown in Figure 3, where the ^1^H-^13^C covalent bonding patterns were observed. The HSQC spectrum provided better resolution for some metabolites such as Glu, Gln, and GSSG (Fig 3C, inset), thereby confirming their assignment.

In addition to metabolite identification, the TOCSY and HSQC analyses provided ^13^C positional isotopomer information for several metabolites in the lung TCA extracts. The ^1^H TOCSY data (cf. Fig. 2B) unambiguously revealed the presence of uniformly ^13^C labeled lactate ([U-^13^C]-lactate) and Ala (([U-^13^C]-Ala) by the ^13^C satellite cross-peak pattern (patterns 1–4 ) of 3-methyl and 2-methine protons of lactate (lactate-H3 and H2) and Ala (Ala-H3 and H2) (traced by dashed green rectangles in Figure 2B) [20]. This pattern is consistent with the enrichment of multiply labeled lactate and alanine of 200–300 fold over the natural abundance levels. The presence of ^13^C-3-Glu, ^13^C-3-Gln, and ^13^C-3-glutamyl residue of oxidized glutathione (Glu-GSSG) was evidenced by the ^13^C satellite cross-peak patterns 9, 10, 14 () and 11–13 () (traced by dashed green lines in Fig. 2B). Patterns 5–8 and 15 denote the presence of ^13^C-2-Glu-GSSG and ^13^C-2-Glu while patterns 16 and 17 indicate the presence of ^13^C-2-Asp.

**Figure 4:**
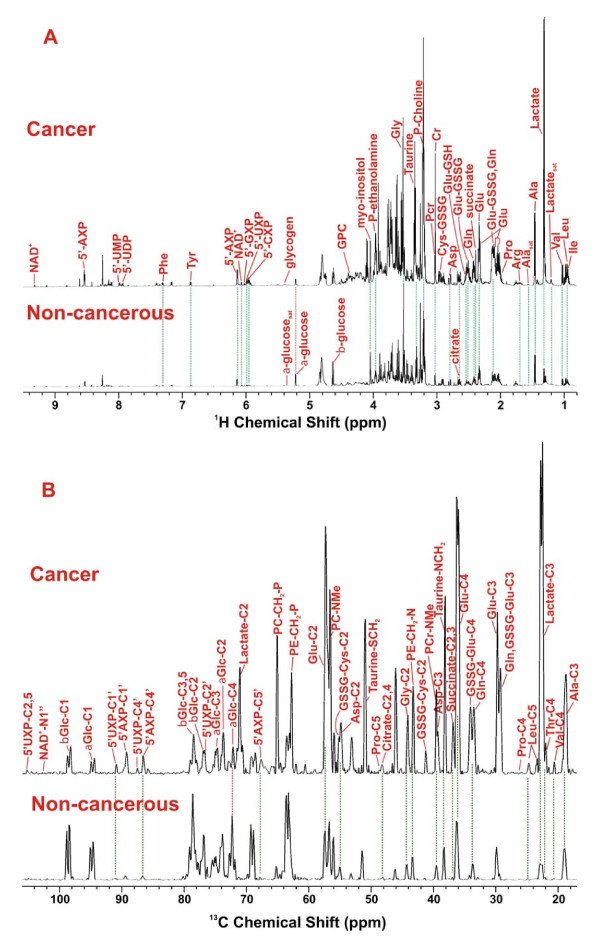
**Comparison of metabolite profiles in TCA extracts of paired non-cancerous and cancerous lung tissues of patient #6**. Metabolites in the 1-D ^1^H NMR (panel A) and ^13^C HSQC projection spectra (panel B) were assigned as in Fig. 2 and 3, respectively. The two sets of spectra were normalized to dry weight and spectral parameters such that the peak intensity of individual resonances is directly comparable. The dashed lines trace metabolites that differed in abundance between cancerous and non-cancerous lung tissues.

The ^13^C-positional isotopomer information obtained from the TOCSY analysis was confirmed by the HSQC analysis, including the positional isotopomers of ^13^C-3-Ala, ^13^C-3-lactate, ^13^C-3-Glu, ^13^C-3-Gln ^13^C-3-Glu-GSSG, ^13^C-2-Glu-GSSG, ^13^C-2-Glu, and ^13^C-2-Asp (cf. respectively Ala-C3, Lactate-C3, Glu-C3, Gln-C3, GSSG-Glu-C3, Glu-GSSG-C2, Glu-C2, and Asp-C2 in Fig. 3B and 4B). In addition, the HSQC data also provided complementary information on isotopomers whose ^13^C satellite cross-peaks were too weak to observe or masked by other cross-peaks in the crowded regions of the TOCSY spectrum [see text and Figure S1 in Additional file 1]. These included selective ^13^C enrichment in C-3-Asp, C-2,3-succinate, C-2,4-citrate, C-1'-ribose-5'AXP, and C-1-α- and -β-glucose (cf. respectively Asp-C3, succinate-C2,3, citrate-C2,4, 5'AXP-C1', and α- and β-Glc-C1, Fig. 3B and 4B).

Based on the metabolite assignment in Figs. 2 and 3, the 1-D ^1^H NMR and ^13^C HSQC projection spectra allowed comparison of metabolite and ^13^C isotopomer profiles in the TCA extracts of paired non-cancerous and cancerous lung tissues. This is illustrated in Figure 4 for patient #6. It is clear in Fig. 4A that the majority of the metabolites (except for glucose) were present at a higher level in the cancerous than in the non-cancerous lung tissue. These also included the ^13^C-labeled isotopomers, [U-^13^C]-lactate and [U-^13^C]-Ala (as denoted respectively by the Lactate_sat _and Ala_sat _resonance in Fig. 4A). The extent of ^13^C enrichment in lactate, Ala (as uniformly labeled species) and Glu (at the C-2 position) was quantified from the respective 2-D ^1^H TOCSY cross-peak patterns (cf. Figure 2B), as previously described [18]. They were consistently higher in the cancer than in the non-cancerous tissue from the same patient, as shown in Table 2. The extent of ^13^C enrichment in glucose (as [U-^13^C]-Glc) for non-cancerous tissues was determined from the 1-D ^1^H spectra (cf. Fig. 4A), which was considerably lower in the cancer than in its non-cancerous counterpart (cf. Table 2). Moreover, increased ^13^C abundance (or ^13^C peak intensity) of most metabolites in cancer relative to non-cancerous tissues was evident in the 1-D HSQC projection spectra in Fig. 4B. Part of the increase in ^13^C abundance from non-cancerous to cancer tissues reflected the difference in total metabolite concentration, which contains 1.1% ^13^C at natural abundance. However, as reasoned in Additional file 1, selective enrichment in ^13^C for a number of metabolite carbons also contributed to the increase in their ^13^C peak intensity. These include C-3-Ala, C-2,3-lactate, C-3-Gln+GSSG, C-2 to 4-Glu, C-4-Gln, C-4-GSSG, C-2,4-citrate, C-2,3-Asp, C-1',4',5'-5'-AXP, and C-1',4'-5'-UXP.

**Table 2:** Enhanced ^13^C enrichment of metabolites in lung cancer tissues relative to their non-cancerous counterpart

Compound	% [U-^13^C]-Ala^A^		% [U-^13^C]-Lactate^A^		% [U-^13^C]-Glucose^A^		% [^13^C-2]-Glu^A^	
	Non-cancerous	Cancer	Non-cancerous	Cancer	Non-cancerous	Cancer	Non-cancerous	Cancer
Mean	1.8	9.1	8.6	15.1	15.5	ND^B^	5.4	8.3
SD^C^	0.9	4.9	4.6	5.4	2.3	-	1.1	1.9
p value^D^	0.009		0.02		-		0.012	

^A ^Values are average of seven patients; % ^13^C-Ala, lactate, and Glu were determined from 2-D ^1^H TOCSY data using appropriate cross-peaks; % [U-^13^C]-glucose was obtained from 1-D ^1^H spectra.

^B ^Not detected or below detection limit; ^C ^Standard deviation; ^D ^from paired t-test

To quantify the ^13^C abundance of metabolites at specific carbon positions, the 2-D HSQC spectra were utilized for the better resolution than the 1-D ^13^C projection spectra (cf. Fig. 4B). This was performed for ^13^C-2,3-succinate of patients #6–10 by integrating the volume of its HSQC cross-peak (cf. Fig. 3C). The relative ^13^C abundance (a measure of selective enrichment) of these two carbons was calculated by normalizing the HSQC peak volume to the total succinate concentration. The relative ^13^C abundance of C-2,3-succinate for tumor tissues (3.6 ± 1.2) was significantly greater than that for non-cancerous tissues (0.6 ± 0.7) with a p value of < 0.01.

### Metabolite &^13^C-Isotopomer profiling of lung tissue extracts by GC-MS

Parallel analysis of the lung TCA extracts by GC-MS served to verify key findings in the metabolite profile obtained by NMR while providing absolute quantification of a subset of metabolites and their ^13^C mass isotopomers. Table 3 shows the quantification of selected metabolites and their total ^13^C enrichment (in excess of natural abundance) by GC-MS. Also shown is the quantification of the m+3 mass isotopomer of Asp (^13^C_3_-Asp or Asp with three of its carbons labeled in ^13^C). For patient #6, the GC-MS data revealed excess total ^13^C enrichment in tumor over non-cancerous tissues for Ala, Asp, Glu, Gln, lactate, citrate and succinate. An enhanced production of ^13^C_3_-Asp in the tumor compared with the paired non-cancerous tissue was also evident. This is consistent with the NMR observation (cf. Fig. 4B) of the buildup of various ^13^C positional isotopomers of metabolites including [^13^C-3]-Ala, [^13^C-2,3]-lactate, [^13^C-2,3,4]-Glu, [^13^C-4]-Gln, [^13^C-2,3]-Asp, [^13^C-2,4]-citrate and [^13^C2,3]-succinate for the tumor, compared with the paired non-cancerous tissues of #6. A similar ^13^C enrichment pattern of Ala, lactate, succinate and citrate in lung tumor tissues was observed in all five patients where ^13^C labeling in these metabolites was sufficient to be quantified by GC-MS. In addition, the enhanced synthesis of ^13^C_3_-Asp in tumor tissues was evident in four of the five patients (Table 3). It should be noted that the fraction of ^13^C_3_-Asp in tumor tissues (0.5 to 1.4%) was far above the natural abundance background (5 × 10^-4^%) (cf. Table 3).

**Table 3:** Total Metabolite and ^13^C-enriched metabolite content of paired non-cancerous and cancerous lung tissues from [U-^13^C]-glucose administered patients^A^

Compound	#6 sqC^B^, grade II		#7 adenoC^B^, grade II		#8 sqC^B^, grade II-III		#9 sqC^B^, grade II		#10 sqC^B^, grade III	
	N^B^	C^B^	N	C	N	C	N	C	N	C
Total Ala	4.67	15.76	6.25	6.44	4.97	9.28	5.14	15.08	5.42	27.34
^13^C-Ala^C^	0.03	**0.28**	0.15	**0.34**	0.09	**0.57**	0.21	**0.79**	0.29	**1.60**
Total Asp	1.32	4.27	3.83	3.09	2.67	1.51	1.94	4.78	2.27	1.66
^13^C-Asp^C^	0.18	**0.59**	0.36	0.44	0.35	0.24	0.21	**0.82**	0.36	0.31
^13^C_3_-Asp^C, D^	< 0.004 (< 0.3%)	**0.058** **(1.4%)**	0.009 (0.2%)	0.005 (0.16%)	0.006 (0.2%)	**0.009** **(0.6%)**	< 0.004 (< 0.2%)	**0.024** **(0.5%)**	0.004 (0.17%)	**0.020** **(1.2%)**
Total Cit^B^	0.60	1.32	1.21	1.18	1.28	1.56	0.77	1.53	0.74	1.37
^13^C-Cit^B, C^	0.03	**0.10**	0.02	**0.05**	0.08	**0.12**	0.03	**0.11**	< 0.004	**0.12**
Total Glu	0.24	4.80	2.96	2.97	1.88	3.25	1.91	4.78	1.82	3.28
^13^C-Glu^C^	0.03	**0.16**	< 0.004	< 0.004	< 0.004	**0.22**	< 0.004	**0.36**	0.10	**0.36**
Total Gln	0.56	7.68	1.41	1.02	0.64	1.32	0.74	2.71	0.79	2.99
^13^C-Gln^C^	0.07	**0.34**	0.07	0.00	0.04	0.00	0.00	**0.21**	0.00	**0.04**
Total Lac^B^	2.66	29.55	11.80	10.62	9.44	17.72	19.52	25.31	8.27	29.67
^13^C-Lac^C^	< 0.004	**0.66**	0.19	**0.63**	0.19	**0.88**	< 0.004	**1.44**	0.03	**0.94**
Total Succ^B^	0.17	1.34	0.50	0.70	0.89	1.84	0.64	1.59	1.14	3.13
^13^C-Succ^C^	0.02	**0.08**	0.01	**0.03**	0.03	**0.11**	0.08	0.14	0.06	**0.19**

^A ^in μmole/g dry weight as determined by GC-MS; the %RSD was generally < 3% in triplicate analyses with the exception of Gln (11%) and ^13^C_3_-Asp (27%).

^B ^sqC: squamous cell carcinoma; adenoC: adenocarcinoma; N: non-cancerous; C: cancer; Cit: citrate; Lac: lactate; Succ: succinate

^C^^ 13^C enrichment in excess of natural abundance; values in **bold **represent enhanced enrichment in cancer over its non-cancerous counterpart

^D^^ 13^C mass isotopomer of Asp with three carbons labeled; detection limit was 0.004 μmole/g dry weight. Values in parentheses are the percentages of ^13^C_3 _isotopomers of the total aspartate. This was much greater than 5 × 10^-4 ^% at natural abundance.

### Quantitative Correlation of ^13^C-Metabolites in human lung tissues

**Figure 5:**
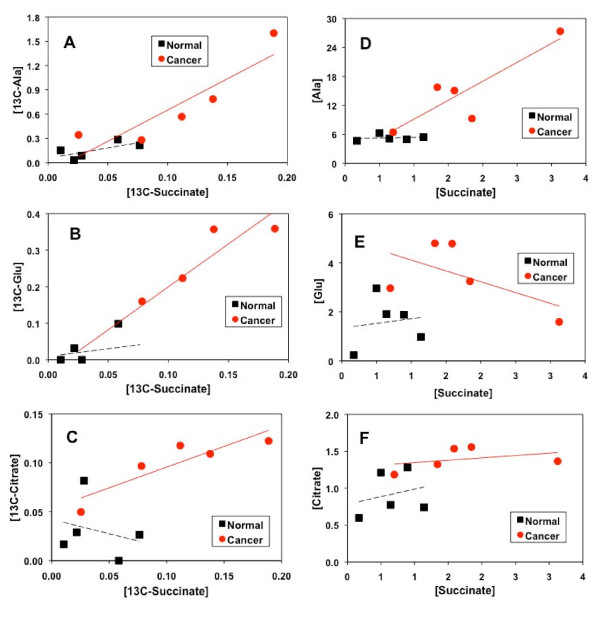
**Relationships between Krebs cycle intermediates and glycolytic products in terms of ^13^C-labeled and total concentrations for lung tumor and non-cancerous tissues resected from patients #6–10**. Metabolite and ^13^C isotopomer concentrations ([metabolite]) were determined from GC-MS analysis as described in Methods. The R^2 ^of the linear fit (solid red lines for cancer and dash black lines for non-cancerous tissues) for these plots are listed in Table 4.

**Figure 6:**
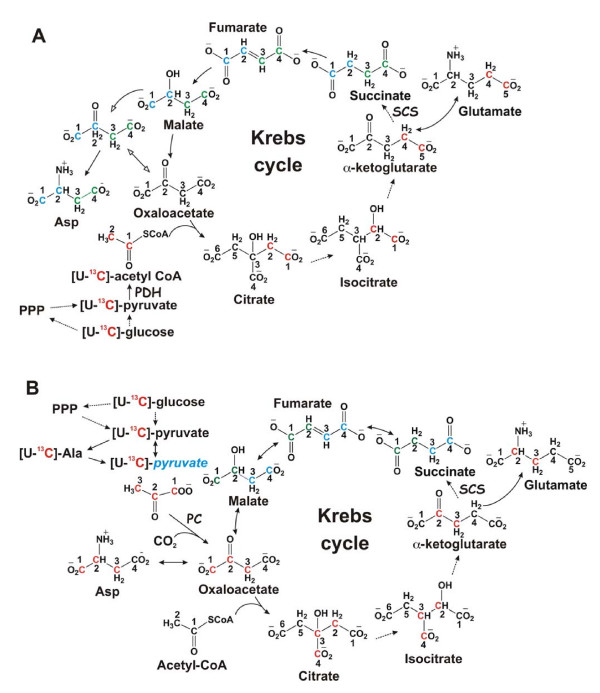
**Expected ^13^C labeling patterns in mitochondrial Krebs cycle intermediates and byproducts with [U-^13^C]-Glc as tracer**. The cycle reactions are depicted without (panel A) or with (panel B) anaplerotic pyruvate carboxylase (PC) reaction and the ^13^C positional isotopomer patterns illustrated are the result of one cycle turn. In the absence of pyruvate carboxylation, Glu is labeled at C4 and C5 positions via the forward cycle reactions while Glu is labeled at C2 and C3 when pyruvate carboxylation is active (panels A and B). The possibility that a separate pool of pyruvate derived from Ala for entry into the Krebs cycle via pyruvate carboxylation is depicted in panel B, along with the contribution of the non-oxidative branch of the pentose phosphate pathway (PPP) to the pyruvate pool. Isotopic scrambling occurs at the symmetric succinate, leading to the redistribution of ^13^C labels into its four carbons, two each at a time (blue and green carbons). Red or blue and green letter C's represent ^13^C labeled carbons before or after scrambling, respectively; blue *pyruvate *denotes a separate pool of pyruvate; solid and dashed arrows denote favorable single and multi-step reactions, respectively; open arrows in panel A delineate ^13^C-labeled OAA after one turn from unlabeled pre-existing OAA.

Utilizing the GC-MS data, pairs of biosynthetically-related ^13^C-labeled metabolites in tumor and non-cancerous tissues were tested for precursor-product relationships, as illustrated in Figure 5 and Table 4. ^13^C-succinate, ^13^C-citrate and ^13^C-Glu are products of the Krebs cycle while ^13^C-Ala is derived from pyruvate, the end product of glycolysis (cf. Figure 6).

**Table 4:** Linear correlation for total or ^13^C-labeled concentrations between pairs of glycolytic- and Krebs cycle-derived metabolites in human lung tissues^A^

	R^2^	
Plots^B^	Non-cancerous	Cancer
[Ala] versus [succinate]	0.025	0.761
[^13^C-Ala] versus [^13^C-succinate]	0.483	0.792
[lactate] versus [succinate]	0.056	0.395
[^13^C-lactate] versus [^13^C-succinate]	0.412	0.374
[Glu] versus [succinate]	0.018	0.343
[^13^C-Glu] versus [^13^C-succinate]	0.072	0.912
[citrate] versus [succinate]	0.061	0.133
[^13^C-citrate] versus [^13^C-succinate]	0.069	0.789

^A ^R^2 ^for the linear regression fit was calculated using Excel for patients #6–10 (n = 5).

^B ^Obtained from GC-MS analysis; [metabolite]: total concentration in μmole/g dry weight of metabolite; [^13^C-metabolite]: μmole/g dry weight of ^13^C-labeled metabolites

A linear correlation in the concentration of ^13^C-succinate with that of ^13^C-Ala, ^13^C-Glu, or ^13^C-citrate (Fig. 5A–C, Table 4) was discernable for lung cancer tissues but not for non-cancerous lung tissues. Also noted was the less significant correlation between ^13^C-succinate and ^13^C-lactate for the lung tumor tissues (Table 4). When total concentrations were plotted, the correlation was even less apparent, particularly for the non-cancerous tissues (Fig. 5D–F, Table 4). This observation underscores the need for acquiring ^13^C-isotopomer data, instead of just steady-state concentrations, to deduce meaningful relationships between transformed products in related pathways. Moreover, in all six plots of Fig. 5, a separation of non-cancerous and tumor tissues was evident, i.e. the tumor and non-cancerous tissues clustered in the high and low concentration quadrants, respectively.

### Gene expression patterns of PC, glutaminase, and krebs cycle dehydrogenases in human lung tumors

The distinct ^13^C labeling patterns in the Krebs cycle metabolites in tumor tissues described above indicates the possibility of altered gene expression in relevant enzymes. This was examined by real-time PCR analysis of key mitochondrial dehydrogenases (DH) along with the anaplerotic pyruvate carboxylase (PC) and glutaminase for tumor and surrounding non-tumorous tissues, as shown in Table 5. Increased expression of the two isoforms of PC gene was evident in patients #6–10 with an average fold change of 3.36 ± 1.13 i.e. significantly higher in tumors (p < 0.01). In contrast, the expression of the other important anaplerotic enzyme gene, glutaminase (GLS) was lower in the tumors relative to the surrounding non-cancerous tissues, with an average fold change of 0.52 ± 0.16. For Krebs cycle DH, there was a substantial decrease of isocitrate DH (IDH) and α-ketoglutarate DH (OGDH) in contrast to a modest activation of malate DH (MDH) expression, while succinate DH (SDH) and fumarate hydratase (FH) showed no statistically significant changes in expression in tumor tissues.

**Table 5:** Changes in gene expression patterns of Krebs cycle and anaplerotic enzymes in lung tumor versus surrounding non-tumor tissues

	Fold Change^A^						
Genes^B^	PC	GLS	IDH3	OGDH	SDH	FH	MDH2
Average^C^	3.36	0.52	0.67	0.58	0.93	1.01	1.53
SD	1.13	0.16	0.13	0.09	0.19	0.23	0.29
p-value^D^	0.03	0.04	0.05	0.01	0.24	0.31	0.04

^A ^Tumor over surrounding non-tumorous lung tissue

^B ^PC: pyruvate carboxylase; GLS: glutaminase; IDH: isocitrate dehydrogenase; OGDH: α-ketoglutarate dehydrogenase; FH: fumarate hydratase; MDH2: mitochondrial malate dehydrogenase

^C ^For patients #6–10

^D ^Obtained from paired *t*-test.

These data differ from a recent report on glioblastoma cells in culture, where glutaminolysis was shown to be significant while pyruvate carboxylation was undetected [13], even though the parent astrocytes have high PC activity [12,32]. Our findings in human NSCLC patients indicate that the activation of either anaplerotic pathway may depend on the tumor cell type. This is consistent with a low glutaminolysis (Gln to lactate) capacity that we found in human NSCLC A549 cells (A.N. Lane, T. W-M. Fan, M.M. McKinney and J.L. Tan, unpublished data), in contrast to the findings for glioblastoma cells.

### PC protein expression patterns in human lung tumors

**Figure 7:**
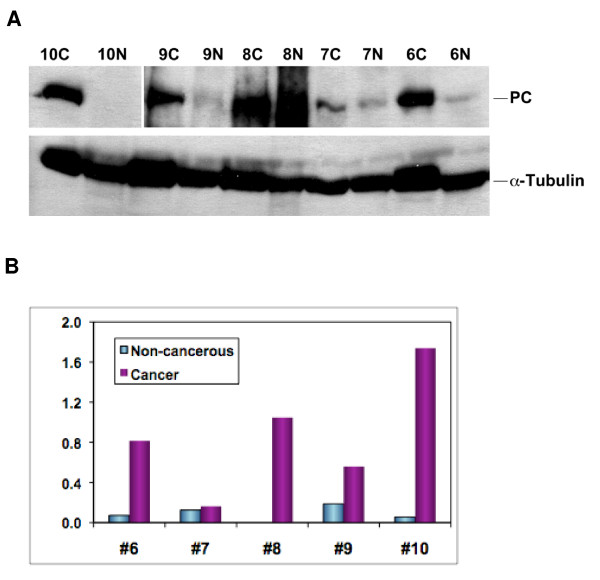
**Western blot analysis of PC protein patterns of paired tumor and non-cancerous tissues from patients #6–10**. Western blotting (panel A) and image analysis (panel B) were performed as described in Methods. Normalized PC response represented PC image density normalized to α-tubulin image density. The non-cancerous tissue of patient #8 had a high interfering background with no discernable PC band in the blot image, which was not quantified. The cancer tissue of patient #10 had a very intense PC band, which along with the PC band of the non-cancerous tissue was quantified using the blot image with 2 min of film exposure, as was the case for the α-tubulin band of all tissues. PC band for the rest of tissues was quantified using the same blot but with 17 min of film exposure. N: non-cancerous; C: cancer; ND: not determined. The data shown is representative of two separate blot analyses.

The *in vivo *^13^C isotopomer profile and gene expression data (see above) indicate increased PC activity in the NSCLC tumors compared with non-tumorous lung tissue. To determine whether PC gene activation leads to an enhanced expression of the enzyme, Western blotting was performed on paired tumor and non-cancerous tissues from the five patients. The PC response normalized to that of α-tubulin is shown in Figure 7B. Lung tumor tissues from patients #6, 9, and 10 exhibited a significant enrichment of PC protein over the non-cancerous counterpart. Patient #8 had a high interfering background over the PC band region for the non-cancerous tissue, which made it difficult to quantify the PC response. The normalized PC response for patient #7 was comparable between the paired non-cancerous and tumor tissues.

## Discussion

### Glycolysis and krebs cycle in human lung tumors is activated

Based on the ^13^C isotopomer analysis by NMR (e.g. Fig. 4, Table 2) and GC-MS (Tables 3, 4), it is clear that human lung tumor tissues exhibited an increased capacity for carbon incorporation from glucose into lactate, Ala, citrate, Glu, succinate, ribosyl moiety of nucleotides, and Asp relative to the surrounding "non-cancerous" lung tissues. The transformation of [U-^13^C]-Glc into [U-^13^C]-lactate and [U-^13^C]-Ala can only occur via glycolysis and to a much lesser extent, the pentose phosphate pathways (PPP), whereas ^13^C-ribose of nucleotides can only be derived from PPP [33]. The enhanced production of these metabolites in tumor tissues provided direct metabolic evidence for the enhancement of the glycolytic capacity [1,34,35]. However, it is unclear whether the increased production of ^13^C-ribose of nucleotides resulted from an enhancement in oxidative and/or non-oxidative branches of the PPP in lung tumor tissues. Further studies with additional tracers (e.g. [^13^C-1,2]-glucose [29]) will be needed to resolve this issue.

The increased conversion of ^13^C carbons from glucose into the Krebs cycle intermediates (citrate and succinate) or related metabolites (Glu and Asp) in tumor compared to non-cancerous tissues (cf. Fig. 4 and Tables 2, 3) is unexpected. We anticipated a reduced and/or disrupted transformation of glucose-carbon into the intermediates of the Krebs cycle, based on the commonly recognized concept of mitochondrial "dysfunction" in cancer [36-38]. Instead, the cycle capacity was not reduced in any of the five lung tumor tissues analyzed. This finding is supported by the enhanced ^13^C incorporation into citrate, succinate, Glu (a surrogate marker of α-ketoglutarate or αKG) and Asp (a surrogate marker of oxaloacetate or OAA) in tumor tissues (Tables 2, 3, 4 and Fig. 4B). The production of these metabolites reflects the operation of the entire cycle (cf. Figure 6), which is also consistent with the significant correlations between ^13^C-labeled citrate, succinate, and Glu in the lung tumor tissues (cf. Fig. 5B, C). It is also interesting to note a stronger relationship between ^13^C-labeled Ala and succinate (Fig. 5A) than that between ^13^C-labeled lactate and succinate in lung tumor tissues. This could imply a separate pool of pyruvate derived from Ala for entry into the Krebs cycle (cf. Fig. 6B). Although unexpected, the combination of activated glycolysis and Krebs cycle rationally explains how the excess demand for energy, NADPH reducing equivalents, and biosynthetic precursors (e.g. Asp for nucleotides, citrate for fatty acyl chains, Glu/Gln for proteins) is fulfilled for tumor growth and proliferation.

### Anaplerotic pathway in human lung tumor is activated

The display of lung tumor tissues in the enhanced production of the [^13^C_3_]-Asp mass isotopomer (cf. Table 3) is intriguing. Given the enhanced expression of PC, this is best explained with the activation of pyruvate carboxylation, i.e. carboxylation of [U-^13^C]-pyruvate to generate [^13^C_3_]-oxaloacetate, which is transaminated to produce [^13^C_3_]-Asp (Fig. 6B). Alternative paths to Asp synthesis from [U-^13^C]-glucose via glycolysis and the first turn of the Krebs cycle leads to two ^13^C labels in Asp, not the three ^13^C-labeled carbons in Asp that was observed (cf. Table 3). For the second turn of the Krebs cycle, [^13^C_3_]-Asp can be produced, provided that [^13^C_2_]-acetyl CoA (derived from [U-^13^C]-pyruvate via PDH) is condensed with [^13^C_2_]-OAA from the first turn. However, the % enrichment of acetyl CoA or [U-^13^C]-pyruvate and [^13^C_2_]-OAA was low, as evidenced by the = 15% enrichment in pyruvate surrogate Ala and lactate and < 8% enrichment in OAA surrogate Asp. Thus, the probability for the condensation of [^13^C_2_]-acetyl CoA in the second turn with [^13^C_2_]-OAA from the first turn is very low (< 1%). In essence, labeled acetyl CoA will always condense with unlabelled OAA, and the labeling pattern of the Krebs cycle intermediates will have the appearance of a single turn, regardless of the actual turn numbers.

Under the low enrichment conditions, the PDH activity produces ^13^C-4,5-αKG (and thus ^13^C-4,5-Glu) and [^13^C_2_]-Asp (m0+2) through the Krebs cycle (cf. Fig. 6A). In contrast, pyruvate carboxylation leads to the production of ^13^C-2,3-Glu via the Krebs cycle, which is distinct from the PDH pathway (cf. Fig. 6B). This distinction in the labeled pattern of Glu provided further metabolic evidence for PC activation in lung tumor tissues. Namely, the ^13^C enrichment of Glu at the C-2 and C-3 positions (Fig. 4B and Table 2) was higher in tumor tissues than its non-cancerous counterpart. Taken together, the ^13^C isotopomer analysis by both NMR and GC-MS revealed the activation of anaplerotic pyruvate carboxylation pathways in NSCLC. It is unclear whether the other anaplerotic pathway, glutaminolysis, is also activated, from the present metabolic data, although the gene expression data indicated otherwise (cf. Table 5).

The metabolomic data were also corroborated by measurements of enhanced gene and protein expression of PC in lung tumors relative to their non-cancerous counterparts. In this case, the ^13^C-isotopomer data was pivotal in directing the interrogations of PC gene expression by RT-PCR and protein analysis by Western blotting. Thus, such metabolomics-edited gene and protein expression analysis (which we have demonstrated for cultured lung cancer cells [22]) is equally useful for uncovering PC up regulation in human patients.

Although previously unknown in human lung cancer, PC activation was recently reported in hepatic tumors in rat [39] and in estradiol-stimulated breast cancer cells [40]. PC expression or suppression has also been respectively associated with enhanced metabolism/proliferation of mammalian cells [41] or inhibition of tumor cell growth [42]. Most recently, PC-induced anaplerotic flux into the Krebs cycle and pyruvate cycling between the cytoplasm and mitochondria was shown to be important in the regulation of glucose-stimulated insulin secretion [30]. These effects of PC are presumably mediated through its ability to replenish the Krebs cycle intermediates, thereby enhancing energy metabolism and fulfilling biosynthetic demands from proliferating cells [41]. From this perspective, it is reasonable to postulate that PC activation may be important for the transformation of lung primary cells into a more highly proliferative state.

## Conclusion

The combined use of [U-^13^C]-glucose tracer and ^13^C isotopomer-based metabolomic analysis by NMR and GC-MS for human lung cancer patients revealed a number of consistent and unexpected features of lung cancer metabolism. The comparison of paired non-cancerous lung and tumor tissues not only enabled consistent conclusion to be made with a small number of samples but also provided the *in vivo *anchor for *in vitro *cell or animal model studies. The general notion of accelerated glycolysis in tumor tissues was supported by the ^13^C labeling patterns of lactate and Ala. More significantly, altered capacity and functioning of the Krebs cycle and anaplerotic pyruvate carboxylation pathway were revealed by the ^13^C labeling patterns of the Krebs cycle metabolites. This is, to the best of our knowledge, for the first time demonstrated *in vivo *for human lung cancers. Further investigations into the molecular regulation of these metabolic traits should provide new insights into lung cancer development and early diagnostic markers, as well as new target(s) for effective therapy. This approach should be generally applicable to other basic and clinical human research.

## Methods

### Patient treatment and sample collection

Lung cancer patients were recruited based on the criteria of surgical eligibility and no history of diabetes. Each patient was consented in accordance with the U.S. HIPAA regulations. The following patient protocol was approved by the Internal Review Board at the University of Louisville. Ten grams uniformly ^13^C labeled glucose ([U-^13^C]-Glc) in sterile saline solution were infused i.v. over a 30 min time period into each patient in the pre-op room approximately 3 or 12 hr prior to resection. Whole blood samples were collected into a vacutainer containing the anticoagulant K_3_EDTA before and after [U]-^13^C-Glc infusion as well as after the surgery. Additional blood samples were collected 3 and 12 hr after the ^13^C-Glc infusion for the 12 hr treatment cases. To minimize metabolic changes, all tissue and blood samples were collected at the operating table with a comparable delay before liquid N_2 _freezing. The blood samples were placed on ice immediately after collection and centrifuged at 4°C at 3,500 × g for 15 min to recover the plasma fraction. All blood samples were aliquoted and flash-frozen in liquid N_2 _within 30 min of collection. Potassium EDTA was used as an anti-coagulant to minimize metabolic artifacts associated with anti-coagulation; it also served to remove the influence of interfering cations such as paramagnetic Fe^3+ ^and Cu^2+ ^for NMR analysis.

All timings from first incision to cutting arteries and veins to the lung were recorded so that the period of ischemia could be determined. Immediately after tissue resection, excess blood was blotted from the tissue, and small pieces of non-cancerous and tumor tissue were cut by the surgeon and freeze-clamped in liquid N_2 _within 5 minutes of removal from the chest cavity. The freezing process arrested metabolic changes almost instantaneously. In all cases, the tumors were well defined so that the extent of the tumor was assessed visually and by palpation. Non-cancerous tissue was removed at least 2 cm from the tumor margin and certified to be tumor-free by trained pathologists. Subsequent pathological evaluation also confirmed tumor status and provided the tumor stage. All samples were stored at -80°C until further processing for analysis.

### Tissue processing and extraction

Frozen tissue samples were pulverized into < 10 μm particles in liquid N_2 _using a Spex freezer mill (Spex CertiPrep, Inc., Metuchen, NJ) to maximize efficiency for subsequent extraction while maintaining biochemical integrity. An aliquot of the frozen powder was lyophilized before extraction for polar metabolites.

Water-soluble or polar metabolites were extracted from lyophilized tissue powders (4–48 mg) in ice-cold 10% trichloroacetic acid (TCA) (v/w minimum 40/1) while leaving proteins, nucleic acids, and polysaccharides behind, as described previously [43]. The extraction was performed twice for quantitative recovery. An aliquot (150 μl) of plasma samples were made to final 10% TCA concentration to precipitate proteins and recover polar metabolites in the supernatant. TCA was then removed from tissue or plasma extracts by lyophilization. The dry pellet was dissolved in nanopure water and two small aliquots were lyophilized for silylation and GC-MS analysis while the remaining bulk was passed through a Chelex 100 resin column (Bio-Rad Laboratories, Inc., Hercules, CA) to neutralize and remove interfering multi-valent cations for NMR analysis.

### NMR analysis

The ^1^H reference standard, DSS (2,2-Dimethyl-2-silapentane-5-sulfonate sodium salt) was added (30 or 50 nmoles) to the TCA extracts of tissue or plasma samples, respectively. NMR analysis of the TCA extracts was performed at 20°C on a Varian Inova 14.1 T NMR spectrometer (Varian, Inc., Palo Alto, CA) equipped with a 5-mm HCN inverse triple resonance pfg cold probe. The following NMR experiments were conducted for the determination of metabolite and ^13^C positional isotopomers: 1-D ^1^H, 2-D ^1^H TOCSY, 2-D ^1^H-^13^C HSQC and HSQC-TOCSY [20,21]. ^1^H and ^13^C chemical shifts of the TCA extracts were referenced to DSS at 0.00 ppm and indirectly to the ^1^H shift, respectively. Various metabolites were identified based on their ^1^H and ^13^C chemical shifts, ^1^H coupling patterns, as well as ^1^H-^1^H and ^1^H-^13^C covalent linkage patterns (acquired from the TOCSY and HSQC experiments, respectively), in comparison with those in our in-house standard database .

For metabolite and ^13^C-isotopomer quantification, selected ^1^H peaks in the 1-D NMR spectra were deconvoluted and integrated using MacNuts software (Acorn NMR, Inc., Livermore, CA). The resulting intensity of peaks of interest was calibrated by the peak intensity of DSS for absolute quantification. Percentage ^13^C abundance of labeled metabolites at specific carbon positions was quantified by integrating appropriate ^13^C satellite peaks in 1-D ^1^H or 2-D TOCSY spectra, as previously described [18,20].

### GC-MS analysis

The same extracts from NMR analysis were subjected to GC-MS analysis for quantifying total and ^13^C-labeled mass isotopomers, as described in full previously [22]. Briefly, the lyophilized extract was derivatized in MTBSTFA (N-methyl-N-[*tert*-butyldimethylsilyl]trifluoroacetamide) (Regis Chemical, Morton Grove, IL) and the tert-butyldimethylslylyl derivatives were separated and quantified on a PolarisQ GC-ion trap MSn (ThermoFinnigan, Austin, TX) equipped with a 50 m × 0.15 mm i.d. open tubular column with 0.4 μm coat BPX-5 (5% phenyl/methyl equivalent) (SGE, Austin, TX). Metabolites were identified based on their GC retention times and mass fragmentation patterns by comparison with those of the standards. Absolute quantification of metabolites was done by calibrating the response of selected ions characteristic of a given metabolite from sample runs with that from standard runs [22]. Relative metabolite abundances were calculated using Xcalibur (ThermoFinnigan, San Jose, CA) or Met-IDEA software [44] to extract peak areas of individual ions characteristic of each component. For Met-IDEA, default program settings for ion trap mass spectrometer were used in the data analysis except for a mass accuracy *m/z *set at 0.001; and a mass range set on either side of the target *m/z *at ± 0.6. For Xcalibur, peak detection, identification, background subtraction, and quantification was performed using parameters custom-tuned to each analyte. Quantification of mass isotopomers was conducted by subtraction of the isotopic profile at natural-abundance (determined empirically from the analyses of standards) from that of the sample. This procedure was repeated for each series of mass isotopomers to arrive at the ^13^C-enriched profiles [22]. In all cases, the pseudo-molecular ion cluster, characteristic of MTBSTFA-derivatives, was used to ensure that true mass isotopomers were quantified.

### Gene expression analysis

From an initial gene microarray analysis of a separate set of six paired tissue samples, a number of statistically significant gene expression differences were discerned between lung cancer tissues and their non-cancerous counterparts. This included an over expression of the pyruvate carboxylase (PC) gene in lung cancer versus paired non-cancerous tissues. Due to sample limitation, the array analysis was not performed for patients #6–10, for which metabolic evidence for PC activation was obtained.

Real time (RT)-PCR was used instead to probe PC gene expression in patients #6–10. Non-cancerous and cancer tissue RNA was isolated using RNeasy minikit (Qiagen) essentially following the manufacturer's instruction. The only modification was that Trizol Reagent (invitrogen), instead of Buffer RLT provided by the kit, was used to disrupt and homogenize the pulverized frozen tissues at the first step of RNA isolation. The modified procedure was found to provide a higher RNA yield and better purity. The integrity of RNA was confirmed by 1% agarose gel electrophoresis. RNA was reverse transcribed into first strand cDNA using oligo(dT)_18 _and SuperScript II reverse transcriptase (Invitrogen). Specifically, 1 μg RNA was added into a 40 μl reaction mixture containing 2 μl 500 μg/ml Oligo(dT)_18_, 2 μl dNTP mix (10 mM each), 8 μl 5× first-strand buffer, 4 μl 0.1 M DTT, 2 μl RNaseOUT™ (40 units/μl) and 2 μl SuperScript II reverse transcriptase (200 units/μl). The reaction mixture was incubated at 42°C for 50 min and then heated at 70°C for 15 min to terminate the reaction.

RT-PCR amplification was performed with SYBR green dye using a Mastercycler ep Realplex 4S (Eppendorf). For each run, 20 μl 2.5 × Real Master Mix (Qiagen), 0.3 μM of forward and reverse primers along with 2 μl first strand cDNA were mixed. The thermal cycling conditions included an initial denaturation step at 95°C for 2 min, 50 cycles at 95°C for 15 s, 55°C for 15 s and 72°C for 20 s. Each reaction was performed in duplicates. The efficiency of the amplification was close to 2.0 (i.e. 100%) for all primer pairs. Relative expression level of each gene was calculated using the Livak method as described previously [45] with 18S ribosomal RNA as the internal control gene. The primer sequences used were designed by Beacon Designer 5.0 (Premier Biosoft International, Palo Alto, CA) as shown in Table 6.

**Table 6:** Forward and reverse primer probes used for RT-PCR of mitochondrial dehydrogenases, pyruvate carboxylase, and glutaminase^A^

Gene/accession no	Forward sequence	Reverse sequence
FH/NM_000143.2	TCTGGTCCTCGGTCAGGTCTG	GACAGTGACAGCAACATGGTTCC
GLS/NM_014905	GCACAGACATGGTTGGTATATTAG	AGAAGTCATACATGCCACAGG
IDH3/NM_005530.2	CAACTGCCCCTTCTCCTATCCC	AGCCCAAGCCTAAGCCCAAG
MDH2/NM_005918.2	CGGAGGTGGTCAAGGCTAAAG	CAGCGGTGTGGAGAAGTAGG
OGDH/NM_002541.2	GTGAGAATGGCGTGGACTAC	CGATTGATCCTGCGGTGATAC
PC/NM_022172	GCGTGTTTGACTACAGTGAG	TCTTGACCTCCTTGAACTTG
SDH/NM_004168.2	CATCGCATAAGAGCAAAGAAC	CCTTCCGTAATGAGACAACC
18S/NR_003286	ATCAGATACCGTCGTAGTTCC	CCGTCAATT CCTTTAAGTTTCAG

^A ^The abbreviations are as in Table 5.

### Western blotting of pyruvate carboxylase

Pulverized and lyophilized lung tissues were extracted twice in chloroform:methanol (2:1) plus 1 mM butylated hydroxytoluene to remove lipids, which interfered with the extraction of PC. The delipidated tissue powder was then extracted in 62.5 mM Tris-HCl plus 2% sodium dodecyl sulfate (SDS) and 5 mM dithiothreitol and heated at 95°C for 10 min. to denature proteins. The protein extract was analyzed by SDS-PAGE using a 10% polyacrylamide gel and separated proteins were transferred to a PVDF membrane (Immobilon™-P, Millipore, Bedford, MA), and blotted against an anti-PC rabbit polyclonal antibody (Santa Cruz Biotechnology, Santa Cruz, CA) overnight at 4°C. PC was visualized with incubation in a secondary anti-rabbit antibody linked to horseradish peroxidase (HRP) (Thermo Scientific, Rockford, IL), followed by reaction with chemiluminescent HRP substrates (Supersignal^® ^West Dura Extended Duration substrate, Thermo Scientific), and exposure to X-ray film. The film was digitized using a high-resolution scanner and the image density of appropriate bands (130 kDa for PC and 50 kDa for α-tubulin) was analyzed using Image J (NIH, Bethesda, MD). The image density of the PC protein band was normalized to that of the α-tubulin protein band.

### Statistical analysis

GC-MS analysis was performed in triplicate and % RSD (relative standard deviation) of analysis was calculated for each reported metabolite (Table 3). Student's paired t-test was performed on each pair of tumor and non-cancerous tissues for the real-time PCR data (Table 5) and for the ^1^H-TOCSY data (Table 2). Linear regression calculations were done for pairs of metabolites in Table 4.

## Abbreviations

HK II: hexokinase II; GAPDH: glyceraldehyde-3-phosphate dehydrogenase; PGK-1: phosphoglycerate kinase 1; LDH-5: lactate dehydrogenase 5; PDH: pyruvate dehydrogenase; PFK-2: phosphofructokinase 2; NSLC: non-small cell lung cancer; HIF: hypoxia-inducible factor; GPx: glutathione peroxidase; GST: glutathione-S-transferase; GSH: reduced glutathione; [U-^13^C]-Glc: uniformly ^13^C-labeled glucose; PPP: pentose phosphate pathway; TCA: trichloroacetic acid; TOCSY: total correlation spectroscopy; GSSG: oxidized glutathione; Cr: creatine; P-choline: phosphocholine; CXP: cytosine nucleotides; UXP: uracil nucleotides; GXP: guanine nucleotides; AXP: adenine nucleotides; HSQC: heteronuclear single quantum coherence spectroscopy; Lactate_sat_,: ^13^C satellites of lactate, alanine, and glucose; Ala_sat_, and glucose_sat_, OAA: oxaloacetate; αKG: α-ketoglutarate; PC: pyruvate carboxylation.

## Competing interests

The authors declare that they have no competing interests.

## Authors' contributions

TWMF performed Western blotting, part of the NMR measurement, NMR data analysis, biochemical interpretation of metabolomic data, participated in experimental design and human specimen collection and processing, and drafted the manuscript. ANL conducted part of the NMR measurement, NMR data analysis and interpretation, participated in experimental design and human specimen collection and processing, and helped to draft the manuscript. RMH designed and performed part of the GC-MS analysis and interpretation, and helped to draft the manuscript. MAF conducted the major part of the GC-MS analysis and helped to draft the manuscript. HG designed and performed quantitative PCR analysis and helped to draft the manuscript. MB performed surgical resection of human lung tissues, participated in experimental design, and helped to draft the manuscript. DMM participated in experimental design and biochemical interpretation, as well as helped to draft the manuscript.

## Supplementary Material

Additional file 1 **Detection of Selective ^13^C enrichment in Specific Carbon Positions of Lung Tissue Metabolites**. The data illustrated how selective ^13^C enrichment at different carbon positions of lung tissue metabolites was determined by a combination of 1-D ^1^H and 1-D ^1^H-^13^C HSQC NMR analysis. Click here for file
