# Supplementary material for: Altered regulation of metabolic pathways in human lung cancer discerned by 13C stable isotope-resolved metabolomics (SIRM)
Source: Mol Cancer. 2009 Jun 26;8:41. doi: 10.1186/1476-4598-8-41 (PMC2717907; doi:10.1186/1476-4598-8-41)
Supplement: Additional file 1 — Detection of Selective 13C enrichment in Specific Carbon Positions of Lung Tissue Metabolites. The data illustrated how selective 13C enrichment at different carbon positions of lung tissue metabolites was determined by a combination of 1-D 1H and 1-D 1H-13C HSQC NMR analysis. [file 1476-4598-8-41-S1.doc]

**Additional file 1**

***Detection of Selective ^13^C enrichment in Specific Carbon Positions of Lung Tissue Metabolites***

Figure S1 illustrates this aspect by comparing the HSQC projection spectra along the ^13^C dimension (Fig. S1A) with the 1-D ^1^H NMR spectra (Fig. S1B) for two each patients infused with [U-^13^C]-Glc (patients #6 and 8) or without (patients #2B and 4B). It should be emphasized that for patients #2B and 4B, the ^13^C resonances in Fig. S1A arose from natural abundance only (ca. 1.1% of total concentration), while some of the resonances for patients #6 and 8 were enriched due to ^13^C label incorporation from [U-^13^C]-Glc, e.g. the 3-carbon of lactate (Lactate-C3).

Selective ^13^C enrichment over natural abundance was revealed as follows. For example, in the ^1^H spectra (Fig. S1B), the peak intensity of the methyl protons attached to the unlabeled 3-carbons of Ala (Ala-H3) and Asp (Asp-H3) was comparable for all four patients, which indicates that the unlabeled Ala and Asp concentrations were comparable. In contrast, the ^13^C peak intensity (or abundance) of the 3-carbon of Ala (Ala-C3) as well as 2 and 3-carbon of Asp (Asp-C2, Asp-C3), Fig. S1A) was proportionally higher for [U-^13^C]-Glc-infused patients (#6 and #8) than for patients without the ^13^C infusion (#2B and #4B). If ^13^C intensities in HSQC spectra were attributed to natural abundance only, then one would expect them to be similar for Ala-C3 and Asp-C2 since Ala and Asp concentrations were comparable. The higher ^13^C abundance of Ala-C3 and Asp-C2 is thus consistent with ^13^C enrichment in these two carbons over the natural abundance for patients #6 and #8.

A similar reasoning applies to the ^13^C enrichment in the 2,3 carbons of succinate (succinate-C23), 2,4 carbons of citrate and the 1’-carbon of the ribosyl residue of adenine nucleotides (5’AXP-C1’) in patient #6. The unlabeled succinate, citrate and AXP levels in #6 were lower than those in #2B and 4B (as estimated from the intensities of 5’AXP-H1’, citrate and succinate ^1^H resonances in Fig. S1B). However, the intensity of the ^13^C resonances for 5’AXP-C1, citrate-C2,4 and succinate-C2,3 in Fig. S1A showed an opposite trend. This pattern of lower concentrations yet higher ^13^C abundance or selective enrichment for Patient #6 – interpretable as higher turnover of 5’-AXP, citrate, and succinate – reiterates the point that steady-state concentrations do not directly track metabolic activity.

In addition, the ^13^C enrichment in the C-1 carbons of α- and β-glucose was clearly demonstrated by the higher intensity and doublet nature of the ^13^C resonances (due to ^13^C-1 and ^13^C-2 spin coupling), in contrast to the lower intensity singlet for #2B and 4B (Fig. S1A). It should be noted that these isotopomers were not evident in the TOCSY spectrum for #6 (Fig. 2) due to the lack of detectable ^13^C satellite cross-peaks. Furthermore, the higher intensity doublet (due to ^13^C-3 and ^13^C-2 spin coupling) of the C-3 resonance of lactate (lactate-C3) for #6 and 8 relative to the less intense singlet for #2B and 4B (Fig. S1A) indicate ^13^C labeling at both 3- and 2-carbons of lactate. The triplet shape of the lactate-C2 resonance is consistent with ^13^C spin coupling of this carbon to those at the 1 and 3 positions. This result verified the presence of [U-^13^C]-lactate, as indicated by the TOCSY (cf. Figure 2B) and GC-MS analysis (cf. Table 3). Finally, the more intense and doublet appearance of Glu-C4 for #6 and 8 indicate ^13^C labeling at this carbon along with C-5 or C-3 of Glu.

**Figure S1**

**
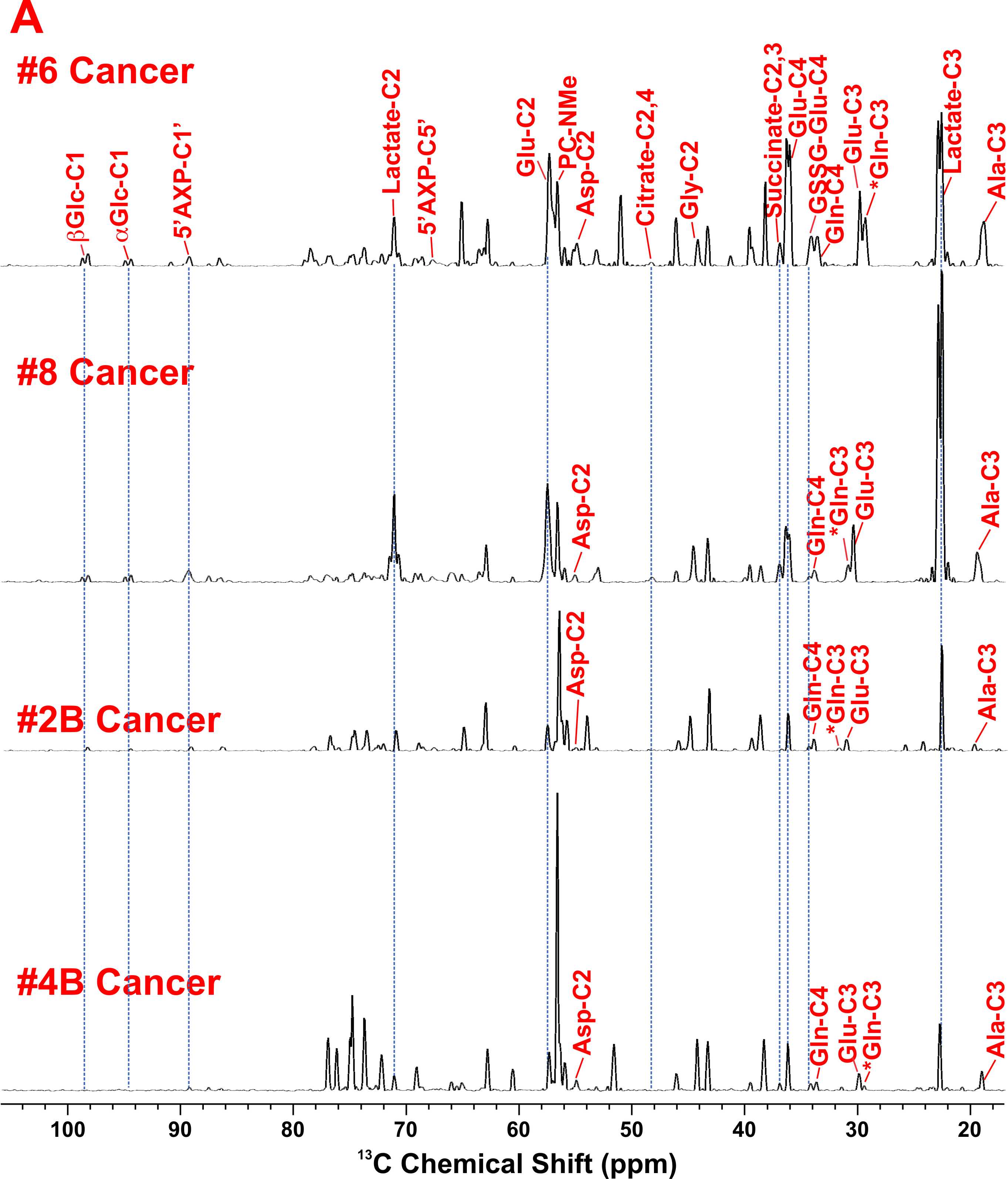
**


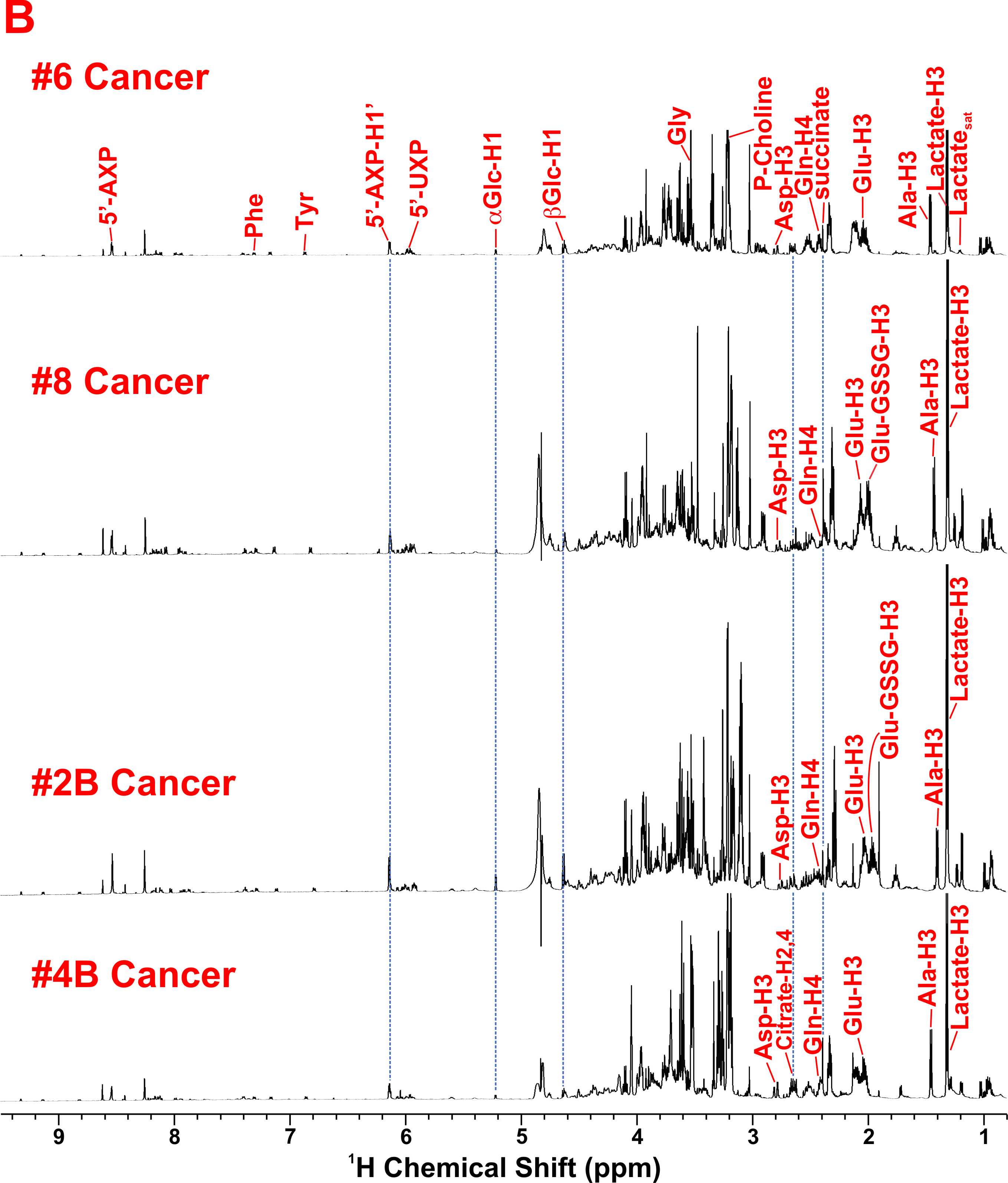


**Figure S1.** Comparison of metabolite profiles in TCA extracts of paired normal and cancerous lung tissues of patient #6. Metabolites in the ^13^C HSQC projection (panel A) and 1-D ^1^H NMR spectra (panel B) were assigned as in Fig. 2 and 3, respectively. The two sets of spectra were normalized to dry weight and spectral parameters such that the peak intensity of individual resonances is directly comparable. The dashed lines trace metabolites that differed in abundance between cancerous and normal lung tissues.
